# Characterization of Mucosal *Candida albicans* Biofilms

**DOI:** 10.1371/journal.pone.0007967

**Published:** 2009-11-24

**Authors:** Anna Dongari-Bagtzoglou, Helena Kashleva, Prabhat Dwivedi, Patricia Diaz, John Vasilakos

**Affiliations:** 1 Division of Periodontology, School of Dental Medicine, University of Connecticut, Farmington, Connecticut, United States of America; 2 Department of Microbiology, University of Texas, Houston, Texas, United States of America; 3 Biothera, Eagan, Minnesota, United States of America; Massachusetts General Hospital, United States of America

## Abstract

*C. albicans* triggers recurrent infections of the alimentary tract mucosa that result from biofilm growth. Although the ability of *C. albicans* to form a biofilm on abiotic surfaces has been well documented in recent years, no information exists on biofilms that form directly on mucosal surfaces. The objectives of this study were to characterize the structure and composition of *Candida* biofilms forming on the oral mucosa. We found that oral *Candida* biofilms consist of yeast, hyphae, and commensal bacteria, with keratin dispersed in the intercellular spaces. Neutrophils migrate through the oral mucosa and form nests within the biofilm mass. The cell wall polysaccharide β-glucan is exposed during mucosal biofilm growth and is more uniformly present on the surface of biofilm organisms invading the oral mucosa. We conclude that *C. albicans* forms complex mucosal biofilms consisting of both commensal bacterial flora and host components. These discoveries are important since they can prompt a shift of focus for current research in investigating the role of *Candida*-bacterial interactions in the pathogenesis of mucosal infections as well as the role of β-glucan mediated signaling in the host response.

## Introduction

Biofilms are well organized microbial communities adhering to an inanimate or living tissue surface [1]. The term mucosal biofilm further denotes a sessile form of microbial growth on mucosal surfaces which can trigger chronic or recurrent infections, usually devoid of cultivable microbes in tissue exudates [2], [3]. *C. albicans* is a commensal colonizer of mucous membranes that can become an opportunistic pathogen, causing common mucosal infections as well as life threatening invasive infections in immunosuppressed patients [4]. It has been hypothesized that biofilm growth of this organism on mucosal surfaces is responsible for the white plaque oral lesions, which are highly diagnostic of pseudomembranous candidiasis [5]. However, its ability to form well organized biofilm communities on oral mucosal tissues has not been documented before. Furthermore, since the oral cavity harbors a vast range of bacterial species [6] it is likely that *C. albicans* interacts closely with the resident bacterial flora during mucosal biofilm formation. However, bacterial-*Candida* co-existence within the oral white plaques in humans or animals has never been demonstrated in situ.

Biofilm cells possess distinct phenotypic characteristics compared to their planktonic counterparts [7]. *C. albicans* biofilms have been shown to have altered composition of their carbohydrate cell walls with an increase in the total content of β-glucans [8]. Polysaccharides such as β-glucans constitute 50–60% of *C. albicans* cell wall and are usually “masked” by a layer of mannan during planktonic in vitro growth [9]. Recent studies, however, have suggested that β-glucans may become “unmasked” during infection in vivo thus allowing dectin-1 recognition and immune activation [10]. Whether β-glucan exposure is associated with a biofilm phenotype or its exposure on the surface of *C. albicans* occurs as a result of the in vivo environment has not been resolved.

There is universal agreement among microbiologists that the study of biofilms is far more difficult than the study of planktonic organisms. In addition to technical challenges associated with the study of abiotic surface biofilms, the study of tissue biofilms is further complicated by the poor accessibility to human tissue samples and/or the lack of faithful animal models of infection. The establishment of adequate models of mucosal biofilm infections is therefore the first step in understanding the mechanisms of biofilm formation on tissue surfaces. In this work, we employed a mouse model of oropharyngeal candidiasis, in which the white plaque lesions were faithfully reproduced, to systematically characterize the composition of mucosal biofilms *in situ*. In some instances the animal work was complemented with experiments using a three-dimensional in vitro model of the human oral mucosa, developed in our laboratory [11], [12].

In this study we hypothesize that *C. albicans* forms complex oral mucosal biofilms involving both bacterial and host components. We provide direct evidence for the first time that epithelial cells, neutrophils and commensal oral bacteria co-exist with *C. albicans* in mucosal biofilms. Furthermore, we demonstrate that β-glucan is present on the fungal cell surface not only during mucosal biofilm development but also during in vitro biofilm growth.

## Results

### Three-Dimensional Structure of Mucosal Biofilms

**Figure 1:**
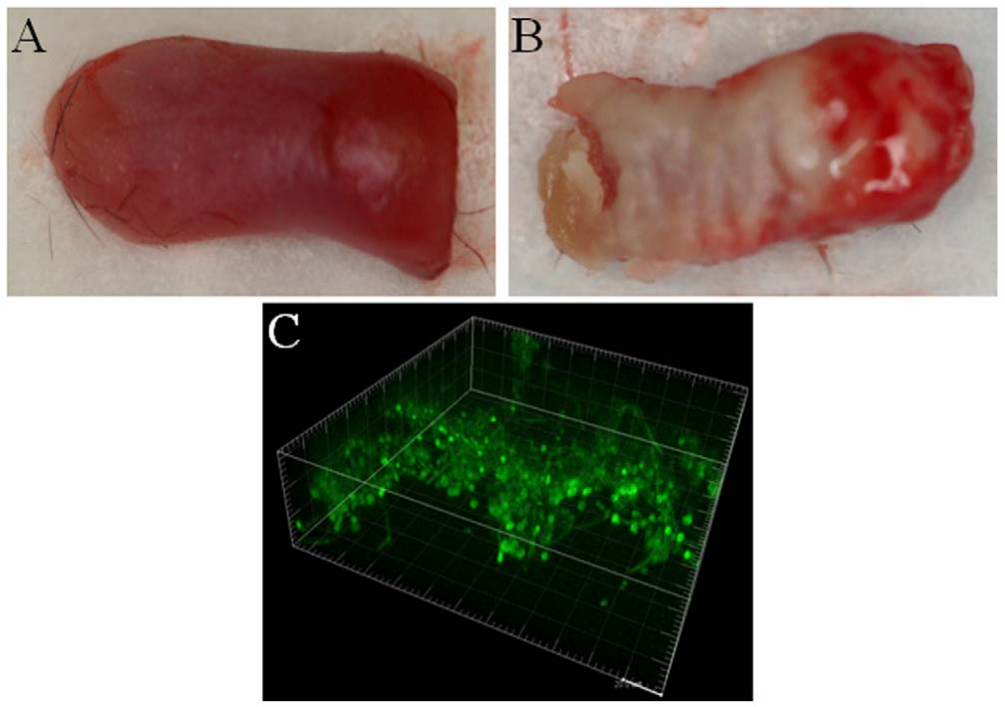
*C. albicans* presence in “white plaque” lesions formed on the tongue of mice with oropharyngeal candidiasis. *C. albicans*-challenged mice were sacrificed after 5 days of oral exposure to the GFP-expressing strain MRL51. Panel A depicts the dorsal aspect of a tongue from an uninfected control. Panel B depicts the white plaque lesions formed on the tongue of an infected mouse. Panel C depicts a three dimensional reconstruction of a live biofilm as visualized via confocal microscopy.

In order to visualize live, fully hydrated biofilms in vivo we infected mice with a GFP-expressing strain of *C. albicans* and examined the white plaques formed on the dorsal surface of the tongue by confocal microscopy (Fig. 1A,B). Confocal imaging followed by 3D reconstruction of live tongue biofilms revealed an architecture that followed the epithelial microanatomical variations of the lingual papillae, forming “valleys” and higher “elevations” of stacking fungal cells (Fig. 1C). We also observed abundant dark areas inter-dispersed among fluorescent organisms, suggestive of extracellular matrix (Fig. 1C).

### Beta-Glucan Distribution in Mucosal Biofilms

**Figure 2:**
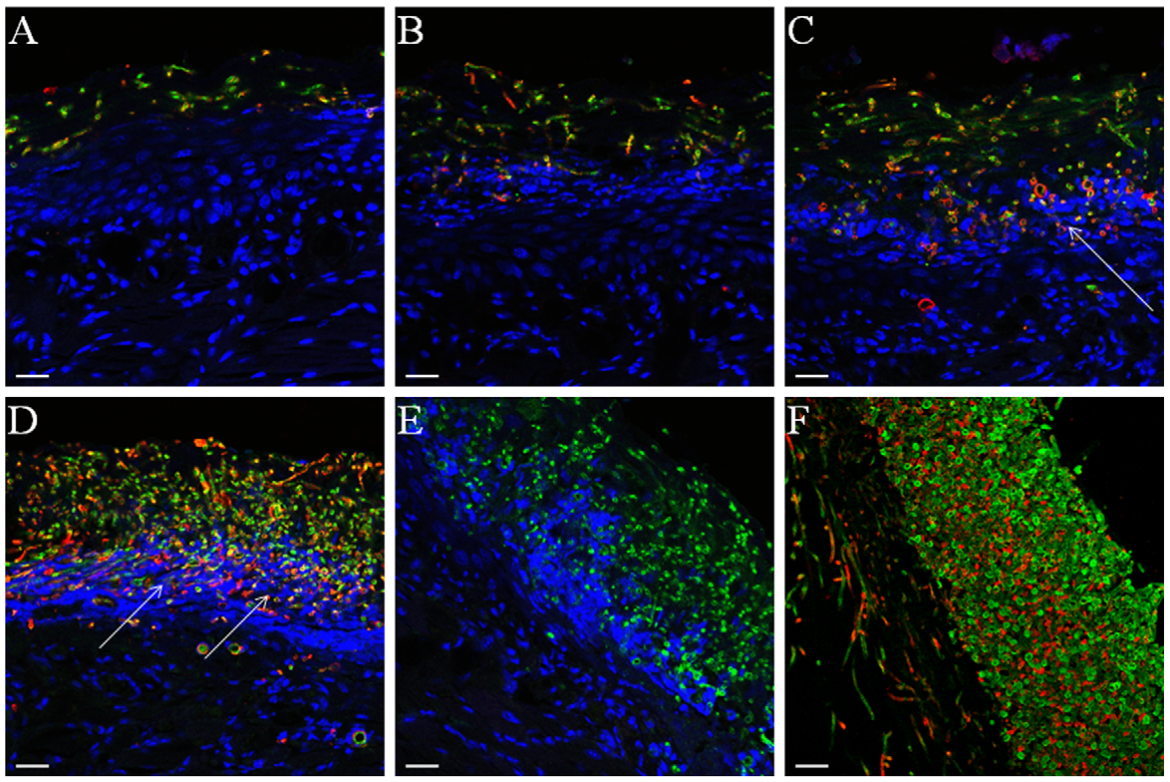
Display of β-glucan during different stages of *C. albicans* mucosal biofilm growth. Panels depict representative confocal images of tissue sections from mice with oropharyngeal candidiasis (A–D) or from a three-dimensional in vitro model of the oral mucosa (F). Panels A–D and F depict sections stained for β-glucan with a BFDiv monoclonal antibody (red), *C. albicans* with a polyclonal anti-*Candida* Ab (green) and counterstained with the nucleic acid stain TO-PRO-3, which stains tissue cells blue. Panel E depicts a section stained with an IgM isotype control antibody for the BFDiv stain. Notice that β-glucan becomes more uniformly present on the surface of fungal cells invading the tongue mucosa (arrows). Scale bar = 20 µm.

Since the total content of β-glucans increases in abiotic surface *Candida* biofilms [8] we decided to characterize its distribution pattern in oral mucosal biofilms. To detect β-glucan we used a monoclonal antibody highly specific for (1→6) branched, (1→3)-β-D-glucans (BFDiv, Biothera) found on fungal cell walls, which does not recognize linear, essentially homogeneous glucans [13]. The specificity of this antibody to *C. albicans* cell wall glucans has been confirmed in other studies [14]. In tissue sections of tongue biofilms β-glucan was immunoaccessible throughout the biofilm mass and its presence was noted on the surface of both yeast and hyphal organisms (Fig. 2A–D). However, the distribution of β-glucan became more uniform on the surface of fungal cells invading the tongue mucosa (Fig. 2C,D). Absence of fluorescent signal in control stains without primary antibody showed that no detectable mouse host IgM was bound to organisms invading the tongue mucosa (not shown). In addition to this control, the specificity of our staining protocol was tested by using a mouse isotype control (IgM) antibody (Fig. 2E).

It has been hypothesized that β-glucan is “unmasked” during the course of infection in vivo due to progressive damage of fungal cells by immune cell attacks [10]. In order to address this possibility, we examined the distribution of β-glucan in mucosal biofilms growing on our three-dimensional in vitro model of the human oral mucosa, which is devoid of an immune cell component [12]. These air-lifted, semi-dry cultures receive media from the basal surface only, thus fungal growth is directed basally, toward the tissue where nutrients are more readily available. As seen in Fig. 2F β-glucan was more abundant in the basal two thirds of the biofilm mass (closer to the tissue surface) and was more uniformly displayed on the surface of the majority of hyphal cells invading into the submucosal compartment. Thus, β-glucan was displayed on the fungal cell surface in the absence of immune cells.

**Figure 3:**
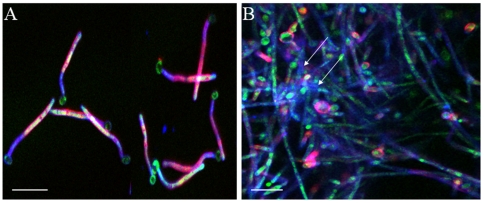
β-glucan and extracellular material staining in *C. albicans* biofilms forming on glass. Panel A depicts a 2h biofilm and panel B depicts a 48h biofilm. Biofilms of the GFP-expressing *C. albicans* strain (green) were stained for β-glucan with a BFDiv monoclonal antibody (red) and the extracellular material was stained with ConA-Alexa 350 (blue). In 2h biofilms there is partial co-localization of the BFDiv mAb and ConA (pink). BFDiv stains parts of the fungal cell, but not the germinating buds, and ConA stains the entire fungal cell surface (3A). In 48h biofilms deposits of cell-dissociated ECM stained with ConA but not with BFDiv (3B, arrows). Scale bar = 20 µm.

**Figure 4:**
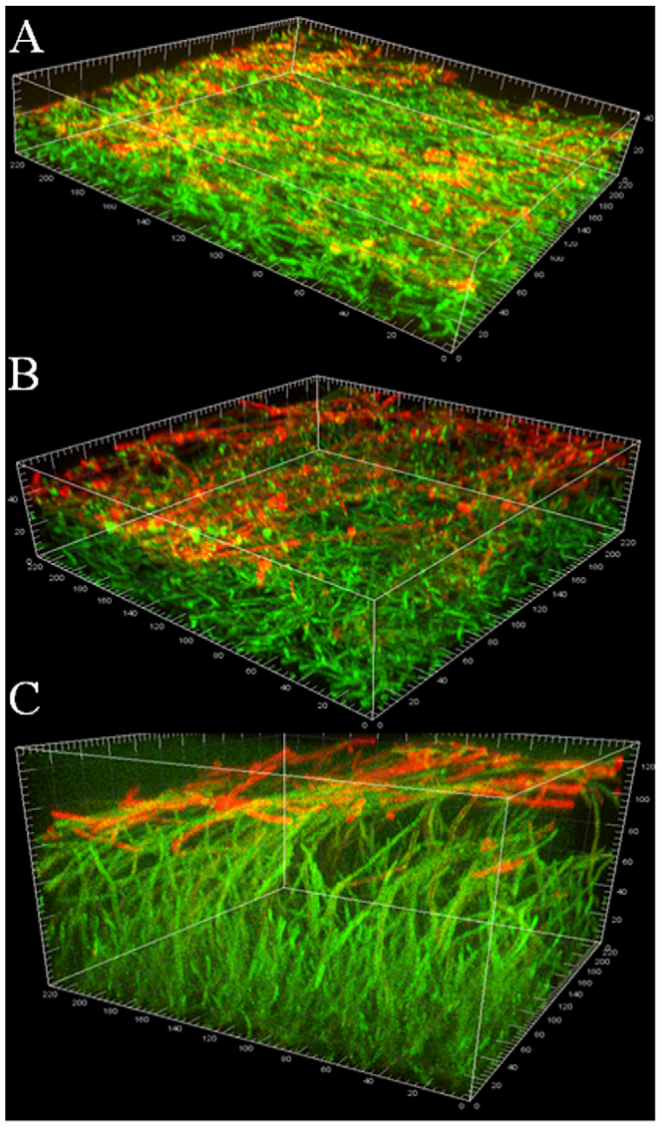
β-glucan and extracellular material staining during *C. albicans* SC5314 in vitro biofilm growth on glass. Panels depict 3D reconstructions of confocal stacks of images of 24h (A), 48h (B) and 72h (C) biofilms of *C. albicans* grown on cover slips and stained for β-glucan with BFDiv mAb (red) and ConA-Alexa 488 (green). Notice that regardless of biofilm thickness β-glucan is localized in the growing end of the biofilm (arrows).

To ascertain whether β-glucan is on the surface of biofilm cells only when growing within a mucosal tissue environment we examined its cellular distribution on abiotic (glass) surface biofilms during different stages of growth. The entire extracellular matrix was visualized with ConA-Alexa 350 (blue, for GFP-expressing strain, figure 3) or ConA-Alexa 488 (green, for strain SC5314, figure 4) and the β-glucan component was visualized using the monoclonal antibody BFDiv, followed by a Cy-3-conjugated secondary antibody (red). This double staining was used to decipher whether the entire or part of the ECM consisted of β-glucan. In early biofilms BFDiv stained parts of the fungal cell, but not the germinating buds, whereas ConA stained the entire fungal cell surface, including the bud (Fig. 3A). Partial co-localization was seen between ConA and BFDiv staining, during early biofilm growth (Fig. 3A, pink). In later stages of biofilm growth, deposits of cell-dissociated ECM stained with ConA (blue) but not BFDiv (Fig 3B, arrows), suggesting that either: a) β-glucan is not present in this diffuse extracellular material; or b) β-glucans in this material are not the highly branched, heterogeneous type of beta glucans recognized by this antibody. Interestingly, in biofilms forming on glass, the cell-wall associated β-glucan was uniformly present at the elongating, unattached end of the biofilm, regardless of biofilm thickness or stage of development (Fig. 4A–C). The striking similarity in the pattern of β-glucan staining in biofilms of variable thickness (30–140 µm, Fig 4A–C) also illustrates that this is not an artifact due to reduced efficiency of penetration of the fluorescent reagent and/or antibody into the biofilm mass. Taken together these observations suggest that β-glucan display on the fungal cell wall during biofilm growth is not specific to tissue biofilms but occurs primarily at the advancing or extending end of biofilms, both in vitro and in vivo.

### Contribution of Host Cell Components in Mucosal Biofilm Structure

**Figure 5:**
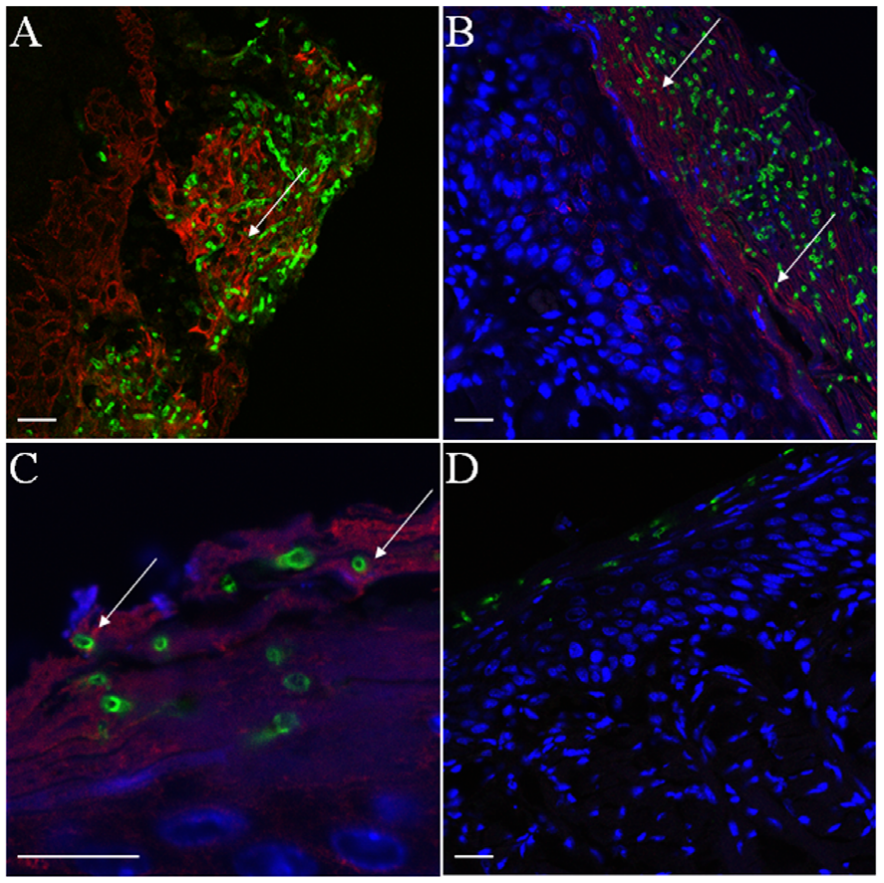
Cytokeratin presence in *C. albicans* biofilms formed on the tongue of mice with oropharyngeal candidiasis. Tissue sections in panels A, B and C were stained with anti-cytokeratin mAb (red) and anti-*Candida* pAb (green). Host cell nuclei were visualized with TO-PRO-3 (blue). Panel D represents an isotype control (IgG) stain. Arrows indicate areas where fungal cells are surrounded by keratin. Scale bar = 20 µm.

Since *Candida* infection triggers a hyperkeratotic response in oral epithelium [15], we hypothesized that keratin originating from desquamating epithelial cells is incorporated in the biofilm mass. We found that a significant proportion of the extracellular material in mouse tongue *Candida* biofilms consisted of keratin associated with desquamating epithelial cells (Fig. 5A). In fact *Candida* was frequently surrounded by keratin squames, which comprised part of the extracellular matrix (Fig. 5B–C, arrows).

**Figure 6:**
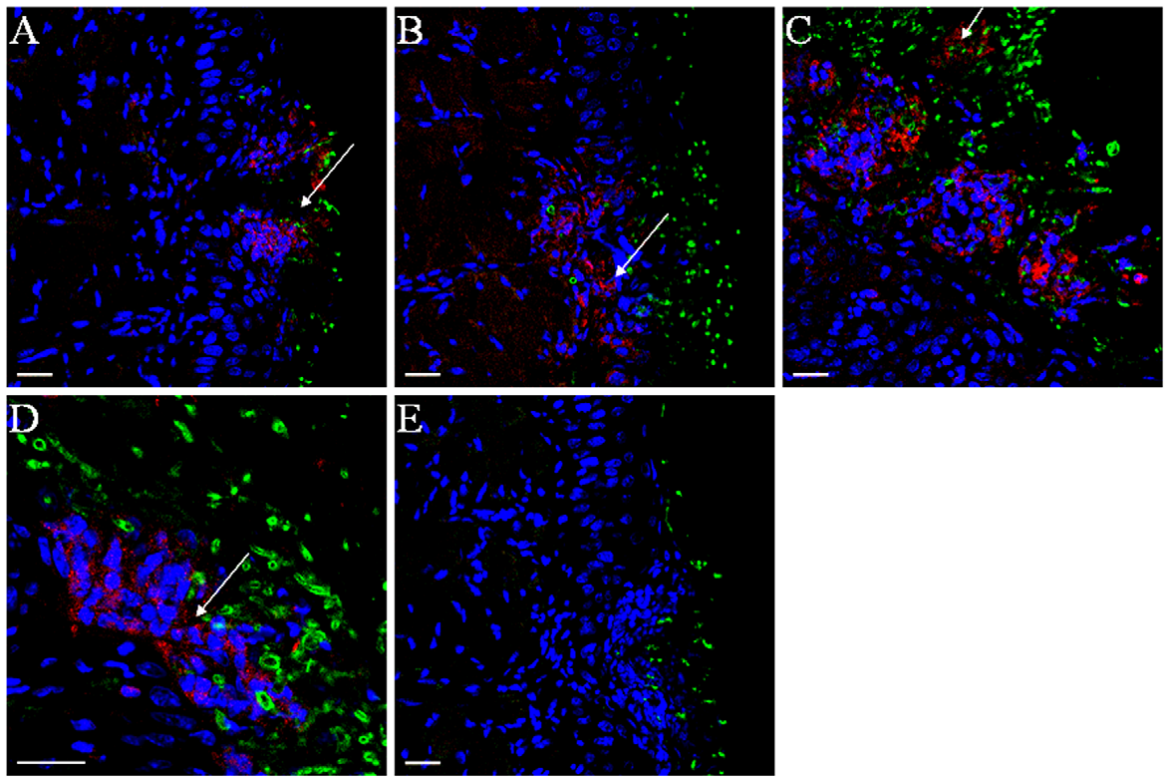
Neutrophils form aggregates in tongue biofilms of *C.albicans*-infected mice. Panels A–D depict tissue sections stained with an anti-mouse neutrophil mAb (red), an anti-*Candida* Ab (green) and the nucleic acid stain TO-PRO-3 (blue). Panel E depicts a confocal image of a negative control stain (primary anti-neutrophil mAb was omitted). Arrows indicate the presence of neutrophils directly juxtaposed to, or within the biofilm mass. Scale bar = 20 µm.

A prominent feature of infections characterized by a soft tissue biofilm is infiltration of infected tissues by neutrophils [16], [17]. Apart from conferring protection at mucosal sites, neutrophils may also become part of the biofilm mass, and their products may serve as a matrix to enhance biofilm formation [18]. We thus hypothesized that neutrophils form part of the biofilm mass on the surface of the tongue in infected animals. To examine the presence of these cells, 3-color CLSM was used to visualize the fungal organisms, epithelial cells and neutrophils. We found that neutrophils formed aggregates juxtaposed to mucosal biofilms (Fig. 6A–C). In sites with thicker biofilms, neutrophils migrated through the entire width of the mucosa, with neutrophil “nests” forming within the biofilm mass (Fig. 6C). No neutrophil aggregates were observed in uninfected animal tissues (not shown).

### Contribution of Oral Bacterial Flora in *Candida* Mucosal Biofilms

**Figure 7:**
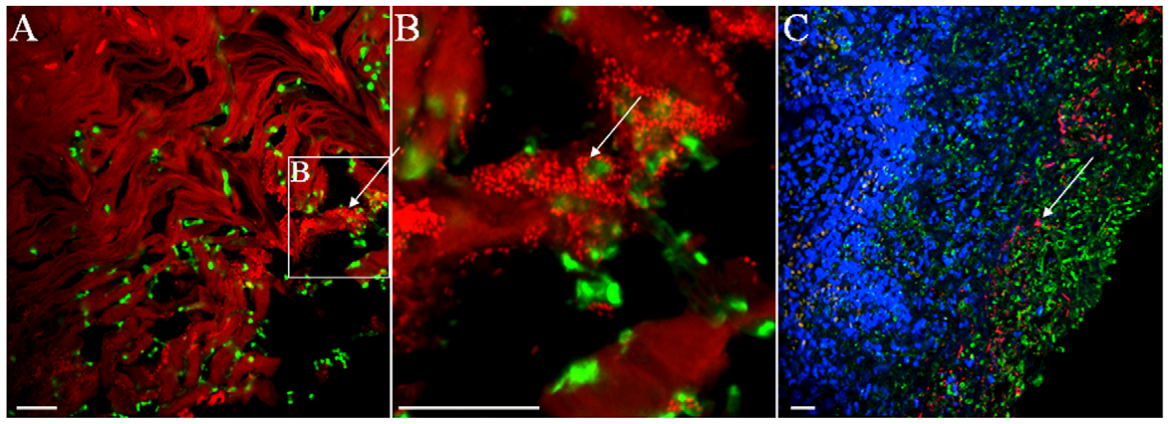
Presence of bacteria in mucosal biofilms of mice with oropharyngeal candidiasis. Panels A and B depict tissue sections stained with an anti-*Candida* pAb (green) and the nucleic acid stain Syto59 (red). Panel B is a 3.5× zoom image of the marked area in Panel A. Notice the close association of *C. albicans* and bacterial cells (arrows). Panel C depicts a tissue section stained with an anti-*Candida* antibody (green), processed for fluorescence in situ hybridization (FISH) with the all bacteria-specific oligonucleotide probe EUB388 (red) and counterstained with the nucleic acid stain Hoechst 33258 (blue). Notice the presence of bacteria (pink) throughout the mucosal biofilms (arrows). Scale bar = 20 µm.

**Figure 8:**
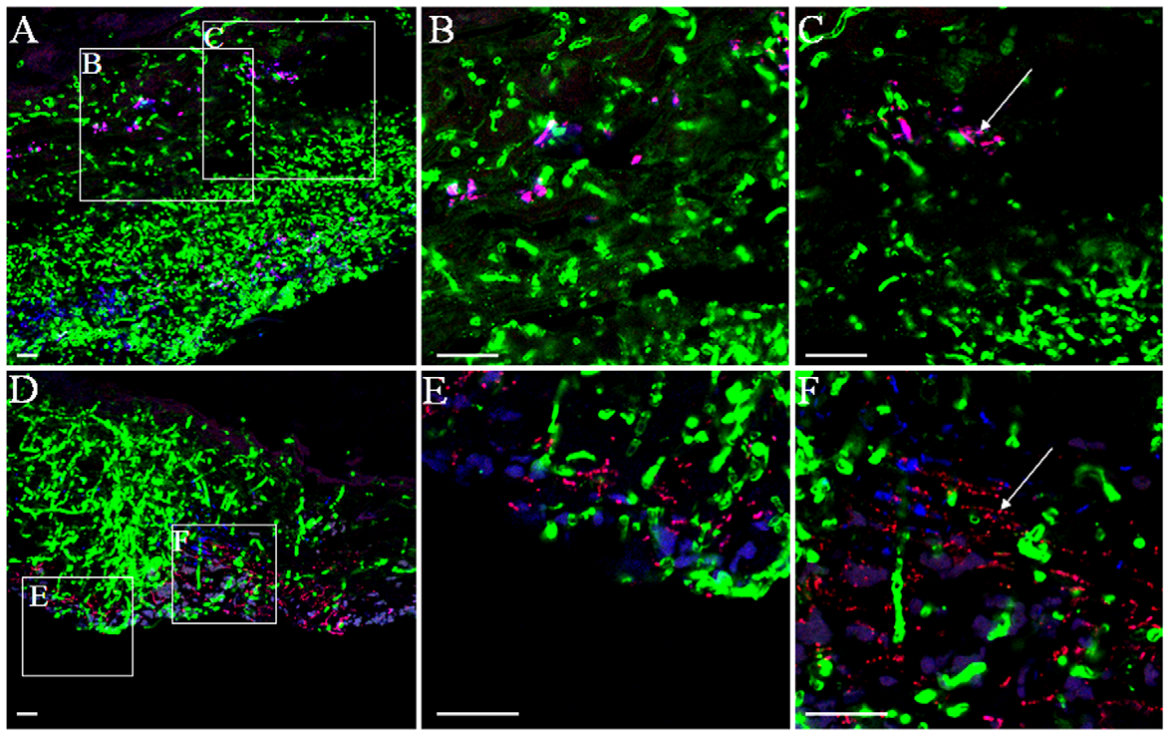
Identification of specific components of bacterial commensal flora in mucosal biofilms of mice with oropharyngeal candidiasis. Panels A-C depict a tongue tissue section stained with an anti-*Candida* antibody (green) and processed for fluorescence in situ hybridization (FISH) with the all bacteria-specific oligonucleotide probe EUB388 (blue) and the *Lactobacillus* and *Enterococcus* sp.-specific probe LAB158 (red). Panels B and C are a 3.5× zoom image of the marked areas in Panel A. Bacteria positive with the LAB158 probe appear pink due to co-localization with the all bacteria specific probe EUB338 (arrows). Panels D and F depict a tongue tissue section stained with an anti-*Candida* antibody (green) and processed for FISH with the all bacteria-specific probe EUB388 (blue) and the *Staphylococcus* sp.-specific probe STA697 (red). Panels E and F are a 3.5× zoom image of the marked areas in Panel D. Bacteria positive with the STA697 probe appear pink due to co-localization with the EUB338 probe (arrows). Scale bar = 20 µm.

Finally, we hypothesized that mucosal fungal biofilms constitute polymicrobial communities containing bacterial species of the resident oral flora. The mouse oral bacterial flora contains about 20 bacterial species which could theoretically contribute to biofilm formation [19]. The most predominant species are *Lactobacillus murinus*, *Staphylococcus* sp. and *Enterococcus faecalis* [19]. We thus developed an immuno-fluorescence in situ hybridization (immuno-FISH) labeling approach to simultaneously visualize *C. albicans* and bacterial commensals in tongue biofilms of mice with oropharyngeal candidiasis. We observed that bacteria were present together with *C. albicans* forming mixed mucosal biofilms as evidenced by positive staining with Syto 59 (Fig. 7A,B) and the all bacteria-specific probe EUB338 (Fig. 7C). Some bacteria were further identified as *Enterococcus/Lactobacillus* sp. (Fig. 8A–C) and *Staphylococcus* sp (Fig. 8D–E) using more specific probes. Tissues from 5 mice stained by this approach were positive for both bacteria and *C. albicans*. Interestingly, mice positive for *Staphylococcus* sp. (4 out of 5) were not positive for *Enterococcus/Lactobacillus* sp. and a single mouse positive for *Enterococcus/Lactobacillus* sp. did not give a positive signal with the *Staphylococcus* sp. probe. It was also observed that most of the bacteria were associated with the apical (not tissue-associated) end of the biofilm, but some bacteria were also seen to invade the tongue epithelium together with *C. albicans*. Using this staining technique we could not identify commensal bacteria adhering to the tongue surface in uninfected animals (not shown), suggesting that *C. albicans* may promote tissue colonization and invasion by normally “innocent” commensals.

## Discussion

In this study for the first time we systematically characterized the structure and composition of biofilms of *C. albicans* growing on the oral mucosa. A similar form of superficial growth and multicellular assembly of *C. albicans* has been previously reported to take place on the stratified squamous epithelium of human ectocervix organ cultures [20]. We conclude that oral mucosal biofilms associated with *C. albicans* infection consist of complex structures containing fungal, bacterial and host cells or cell-derived products.

Abundant glucans can be extracted from the extracellular material of *Candida* biofilms [21] and a glucan-cross-linked protein has been shown to be critical in biofilm development [22], therefore it was not surprising that β-glucan was readily identifiable within mucosal biofilms. The prominent staining pattern of β-glucan in cells invading the oral mucosa was consistent with that seen on *C. albicans* cells invading the kidneys of systemically infected rats or mice [10], [14]. Cell surface localization of β-glucan in vivo was not morphotype-specific, again consistent with recent studies in systemically infected mice, in which a different anti- β-glucan monoclonal antibody was used [10]. However in our studies, the β-glucan staining pattern was similar in vitro and in vivo, since it was primarily localized at the active site of biofilm growth, which indicates that it may be a characteristic of a growing or immature biofilm phenotype and not a result of exposure to the in vivo environment as it was previously suggested [10]. Since β-glucan plays a role in the immune recognition of *Candida* [23], [24] and neutrophils are clustered both adjacent and within the biofilm mass, access to cell wall β-glucan may play an important role in their activation via dectin-1-mediated signaling [14], [25]. Therefore the functional role of this receptor-ligand interaction in the context of mucosal or catheter-associated biofilm infections needs to be further investigated.

These studies for the first time shed light on the complexity of oral mucosal biofilms formed by *C. albicans*. Our findings support the idea that the composition of mucosal biofilms is inherently more complex than abiotic surface biofilms [18], [26] since host cells and the resident bacterial flora form part of the biofilm mass. The identification of bacteria within the biofilm mass in situ was not unexpected since bacteria are often co-isolated with *Candida* from ulcerative lesions of the oral mucosa in humans [27]. However for the first time we provide direct evidence for complex polymicrobial communities in pseudomembranous lesions using an experimental model of *Candida* infection. The relative contribution of these communities to the pathogenesis of these lesions is unknown, with one report suggesting bacterial involvement [28]. Thus novel hypotheses can arise from our discovery-driven work, one of the most intriguing being the potential synergistic relationship between *C. albicans* and the oral bacterial flora, since bacterial adhesion and invasion of the tongue mucosa was noted only in the *C. albicans*-infected animals. Although antagonistic relationships between *C. albicans* and prokaryotes have been described [29], [30], [31], little is known about potential mechanisms of enhancement of bacterial virulence by *C. albicans* [32].

In conclusion, our studies provide valuable new insights that will lead to novel hypothesis-driven research that will further the understanding of mucosal biofilm infections.

## Methods

### Ethics Statement

The study was approved by the University of Connecticut Health Center Animal Care Committee. Animals were monitored daily for distress. Given that the oral cavity is readily accessible, lesions are detected relatively early in their onset and animals are euthanized after lesion formation before visible distress/behavior signs are observed.

### Organisms

Strain SC5314, which was originally isolated from a patient with invasive disseminated candidiasis [33], and displays a virulent phenotype in several oral mucosal models [11], [34], was used to study in vivo and in vitro biofilm growth. A GFP tagged strain (MRL51), derived from strain SC5314 was generously supplied by Dr. A. Mitchell (Carnegie Mellon University) and used for live biofilm visualization. The strains used in this study showed similar growth rates in YPD liquid medium, as determined by direct cell counts of yeast cells, or by the XTT assay [35]. The organisms were routinely grown in YPD broth (Difco Laboratories, Detroit, MI) at 25°C overnight, then washed in PBS and counted in a hemacytometer.

### Mouse Model of Oropharyngeal Candidiasis

A mouse model of pseudomembranous oral candidiasis was used in order to characterize mucosal biofilms in vivo. In these experiments 6–8 week old female C57BL/6 mice were infected with strain SC5314 or its GFP-tagged derivative (5 mice per group). One day prior to infection mice were immunosuppressed by subcutaneous injection with cortisone acetate (225 mg/kg) dissolved in 200 µl PBS containing 0.5% Tween-20. To deliver *C. albicans* challenge mice were anaesthetized by an intramuscular injection of ketamine: xylazine (90–100 mg/kg and 10 mg/kg of body weight, respectively) and a small cotton pad soaked with 100 µl of *C. albicans* cell suspension (6×10^8^ yeast/ml) was used to swab the entire oral cavity. The swab was left for 2 h under the tongue and was removed before the animals awoke. This procedure was repeated 2 days later and mice were sacrificed after 5 days of total exposure to *C. albicans*. During this time period animals were also given drinking water containing a daily-fresh suspension of *C. albicans* (6×10^6^ yeast organisms/ml) to maintain high oral carriage loads throughout the experimental period, since oral carriage is one of the most frequently identified risk factors of human oral infection [36], [37]. At the end of each infection period, tongues were dissected, formalin-fixed and embedded in paraffin. Five µm sections were stained for immunofluorescence and visualized by laser confocal scanning microscopy (LCSM). To observed live, hydrated mucosal biofilms produced by the GFP-expressing strain, unfixed intact pieces of tongues with biofilms were mounted on glass-bottom Petri dishes (MatTek Corp.; Ashland, MA) in PBS-2% glucose and examined in an inverted confocal microscope.

### Three-Dimensional Model of the Human Oral Mucosa

To investigate in vitro mucosal biofilm formation by *C. albicans* we used a three-dimensional model of the oral mucosa as previously described [12]. This system is composed of 3T3 fibroblasts embedded in a biomatrix of collagen type I, overlaid by a multilayer of well-differentiated oral epithelial cells (OKF6/TERT-1). *C. albicans* cells (1×10^6^ yeast cells) were added to the cultures apically in 100µl of airlift medium [12] without FBS and antibiotics. After 24–48 hours of co-culture infected mucosal tissues were formalin-fixed and embedded in paraffin. Five µm sections were stained by immunofluorescence and visualized by LCSM.

### Abiotic Surface Biofilms

For growth of biofilms on abiotic surfaces yeast cells of strain SC5314 or its GFP-tagged derivative were seeded on FBS-coated glass cover slips in 12 well plates at a density of 1×10^7^ cells/well. After growth in YNB medium containing 0.5% glucose at 37°C for 2–48 hours, they were stained with anti β-glucan monoclonal antibody (as described below) and ConA conjugated with Alexa Fluor 488 (for strain strain SC5314) or Alexa Fluor 350 (for GFP-expressing strain, both dyes at 40 µg/ml, Invitrogen, Carsbad, CA). Cover slips were mounted on slides or glass bottom dishes, and biofilms were observed with a Zeiss LSM 510 NLO/FSM microscope. Z-sections were collected and reconstructed into 3D images using the IMARIS software (Bitplane, Inc., Saint Paul, MN).

### Immunofluorescence

Tissue sections from formalin-fixed paraffin-embedded samples were incubated for 1 hour with monoclonal antibodies against β-glucan (22 µg/ml, clone 10C6, Biothera, Eagan, MN), monoclonal anti-pan cytokeratin (1.2 µg/ml, clone AE1/AE3, Dako), or anti-neutrophil monoclonal antibody (1 µg/ml, clone NIMP-R14, Santa Cruz Biotechnology, Santa Cruz, CA). For cytokeratin and neutrophil staining, antigen retrieval was performed by heating sections at 96°C for 30 minutes with Target Retrieval Solution (DakoCytomation, Carpinteria, CA) prior to incubation with primary antibodies. This was followed by a 30-minute incubation with the appropriate Cy-3-conjugated secondary antibody (goat F(ab)′_2_ anti-mouse IgM, goat anti-mouse IgG, or Goat anti-Rat IgG, respectively, all from Jackson Immunoresearch, West Grove, PA) and FITC-conjugated rabbit anti-*Candida* polyclonal antibody (40 µg/ml, Meridian Life Science, Cincinnati, OH). Blocking and staining for cytokeratin was done using the MOM system (Vector; Burlingame, CA). Lastly, TO-PRO-3 staining of cell nuclei was performed (2µM, Invitrogen, Carsbad, CA) for 30 minutes in some samples. Stained sections were examined with a Zeiss LSM 510 NLO/FSM confocal scanning laser microscope (Carl Zeiss Microimaging, Inc.; Thornwood, NY) equipped with argon (488nm) and HeNe (543nm and 633nm) lasers, using a water immersion C-Apochromat 40× objective (NA1.2).

### Fluorescence In Situ Hybridization

Formalin-fixed tissue sections were deparaffinized and stained for 1 h with a FITC-labeled anti-*Candida* polyclonal antibody (Meridian Life Science, Cincinnati, OH). Slides were then washed with PBS and permeabilized with lysozyme (70,000 U/mL in 100 mM Tris/HCl pH 7.5, 5 mM EDTA) for 10 min at 37°C in a humid atmosphere. Samples were then dehydrated in a series of ethanol washes (50, 80 and 100% ethanol; 3 min each) and exposed to 25 µL of hybridization buffer (0.9 M NaCl, 20 mM Tris/HCl pH 7.5, 0.01% sodium dodecyl sulfate and 25% formamide) containing 10 ng/µL of probe. Slides were incubated at 46°C for 90 min in a humid atmosphere and washed for 15 min at 48°C in washing buffer (20 mM Tris/HCl pH 7.5, 5 mM EDTA, 0.01% sodium dodecyl sulfate and 159 mM NaCl). After the immuno-FISH procedure some samples were counter-stained with the nucleic acid stain Hoechst 33258 (Invitrogen, Carsbad, CA). In samples where no FISH was performed, slides were stained with anti-*Candida* antibody and the nucleic acid stain Syto 59 (Invitrogen). The oligonucleotide probes used were purchased from Eurofins MWG/Operon and included the EUB338 probe specific for bacteria [38] labeled with either Alexa 546 or Alexa 633, the LAB158 probe specific for *Lactobacillus* and *Enterococcus* sp. [39] labeled with Alexa 546, and the STA697 probe specific for *Staphylococcus* sp. [40] labeled with Alexa 546. The specificity and efficiency of all probes at 25% formamide was first tested in vitro with laboratory strains representing the target genera and unrelated control species. All samples were observed with a 40× 1.3 NA oil-immersible lens on a Zeiss LSM 510 NLO/FSM confocal scanning laser microscope.
